# Insulin Activates Vagal Afferent Neurons Including those Innervating Pancreas via Insulin Cascade and Ca^2+^ Influx: Its Dysfunction in IRS2-KO Mice with Hyperphagic Obesity

**DOI:** 10.1371/journal.pone.0067198

**Published:** 2013-06-26

**Authors:** Yusaku Iwasaki, Kenju Shimomura, Daisuke Kohno, Katsuya Dezaki, Enkh-Amar Ayush, Hajime Nakabayashi, Naoto Kubota, Takashi Kadowaki, Masafumi Kakei, Masanori Nakata, Toshihiko Yada

**Affiliations:** 1 Division of Integrative Physiology, Department of Physiology, Jichi Medical University School of Medicine, Shimotsuke, Tochigi, Japan; 2 Health Science Service Center, Kanazawa University, Kanazawa, Ishikawa, Japan; 3 Department of Diabetes and Metabolic Diseases, Graduate School of Medicine, University of Tokyo, Tokyo, Japan; 4 First Department of Medicine, Saitama Medical Center, Jichi Medical University School of Medicine, Saitama, Saitama, Japan; 5 Department of Developmental Physiology, Division of Adaptation Development, National Institute for Physiological Sciences, Okazaki, Aichi, Japan; University of California, Los Angeles, United States of America

## Abstract

Some of insulin’s functions, including glucose/lipid metabolism, satiety and neuroprotection, involve the alteration of brain activities. Insulin could signal to the brain via penetrating through the blood-brain barrier and acting on the vagal afferents, while the latter remains unproved. This study aimed to clarify whether insulin directly regulates the nodose ganglion neurons (NGNs) of vagal afferents in mice. NGs expressed insulin receptor (IR) and insulin receptor substrate-2 (IRS2) mRNA, and some of NGNs were immunoreactive to IR. In patch-clamp and fura-2 microfluorometric studies, insulin (10^−12^∼10^−6^ M) depolarized and increased cytosolic Ca^2+^ concentration ([Ca^2+^]_i_) in single NGNs. The insulin-induced [Ca^2+^]_i_ increases were attenuated by L- and N-type Ca^2+^ channel blockers, by phosphatidylinositol 3 kinase (PI3K) inhibitor, and in NGNs from IRS2 knockout mice. Half of the insulin-responsive NGNs contained cocaine- and amphetamine-regulated transcript. Neuronal fibers expressing IRs were distributed in/around pancreatic islets. The NGNs innervating the pancreas, identified by injecting retrograde tracer into the pancreas, responded to insulin with much greater incidence than unlabeled NGNs. Insulin concentrations measured in pancreatic vein was 64-fold higher than that in circulation. Elevation of insulin to 10^−7^ M recruited a remarkably greater population of NGNs to [Ca^2+^]_i_ increases. Systemic injection of glibenclamide rapidly released insulin and phosphorylated AKT in NGs. Furthermore, in IRS2 knockout mice, insulin action to suppress [Ca^2+^]_i_ in orexigenic ghrelin-responsive neurons in hypothalamic arcuate nucleus was intact while insulin action on NGN was markedly attenuated, suggesting a possible link between impaired insulin sensing by NGNs and hyperphagic obese phenotype in IRS2 knockout mice These data demonstrate that insulin directly activates NGNs via IR-IRS2-PI3K-AKT-cascade and depolarization-gated Ca^2+^ influx. Pancreas-innervating NGNs may effectively sense dynamic changes of insulin released in response to nutritional states. These interactions could serve to convey the changes in pancreatic and systemic insulin to the brain.

## Introduction

The vagal afferents, as well as the blood-brain barrier (BBB), serve as the anatomical and functional routes for signaling from the periphery to the brain. It has been shown that the intestinal hormones released upon meal intake, including cholecystokinin (CCK), glucagon-like peptide 1, and peptide YY, act on the vagal afferents to suppress food intake [1].

Insulin, a major hormone released from the pancreas upon food intake, is known to influence peripheral organs and central nerves system (CNS) to regulate a variety of physiological functions, including glucose/lipid metabolism [2], [3], reduction of food intake [4], [5], and growth and differentiation of the body and brain [6], [7]. Moreover, insulin resistance is implicated in learning disorder and Alzheimer’s disease [8], [9].

Up to present, it has been reported that 0.046% of peripheral insulin penetrates BBB [10], that insulin receptor (IR) is expressed in the brain [11], and that neuron-specific deletion of IRs in the brain alters fuel metabolism, reproduction, and hepatic glucose production [11] as well as inducing diet-sensitive obesity and female-selective hyperphagia [12]. Evidences shown in these reports support that insulin exerts the central action at least partly via its direct interaction with IRs on the neurons in the brain. In contrast, whether insulin induces the central effects partly via interacting with vagal afferents remains to be clarified. It has been reported that the local branches of vagal afferents that innervate particular organs/tissues play an important role in sensing/conveying the local information to the brain [13].

In the present study, we aimed to clarify whether insulin directly acts on vagal afferent neurons, and if so, to identify the intracellular signal transduction and the neurotransmitter in the insulin-responsive neurons. For this, we measured membrane potential and cytosolic Ca^2+^ concentration ([Ca^2+^]_i_) in the vagal afferent neurons isolated from the mouse nodose ganglion (NG). Furthermore, whether a specific subpopulation of NG neurons that innervate the pancreas responds to insulin was examined. We also explored whether the NG neurons can sense the insulin levels that change under fasting vs. fed conditions and upon stimulation with insulin secretagogue.

We here show that insulin induces depolarization and increases [Ca^2+^]_i_ through the signaling cascade of IR, insulin receptor substrate-2 (IRS2) and phosphatidylinositol 3 kinase (PI3K) in NG neurons including those containing cocaine- and amphetamine-regulated transcript (CART) peptide and those innervating the pancreas. We also show results to support that NG neurons can sense the change of insulin in the pancreas in response to food intake and insulin secretagogue sulfonylurea.

## Materials and Methods

### Materials

CCK-8 (26–33, sulfated form), ω-conotoxin GVIA, ghrelin (rat) were purchased from Peptide Institute (Osaka, Japan). Insulin (porcin), capsaicin (CAP), verapamil hydrocholoride, LY294002 and glibenclamide were obtained from Sigma (MO). U0126 was obtained from Cell Signaling Technology (MA).

### Animals

Male ICR mice aged 1∼3 months, C57BL/6J mice (2∼5 months), Wistar rats (2 months) were purchased from Japan SLC (Shizuoka, Japan). The male IRS2 knockout mice (IRS2-KO mice, 2∼5 months) on the background of C57BL/6J were provided by Drs. N. Kubota and T. Kadowaki at University of Tokyo. The animals were housed for at least 1 week under conditions of controlled temperature (23±1°C), humidity (55% ±5%), and lighting (light on at 7∶30 and off at 19∶30). Food and water were available *ad libitum* (*ad lib*). Procedures of animal experiments were approved by the Animal Care and Use Committee of Jichi Medical University.

### Isolation of Single Neurons from Nodose Ganglia

Single NG neurons were isolated as previously reported [14]. Briefly, nodose ganglia from mice were treated for 20 min at 37°C with 0.5 mg/mL collagenase Ia (Sigma), 0.5 mg/mL dispase II (Roche, Basel, Swiss), 15 µg/mL DNase II type IV (Sigma), and 0.75 mg/mL bovin serum albumin (BSA, Sigma) in HEPES-buffered Krebs-Ringer bicarbonate buffer (HKRB) composed of (in mM) 4.7 KCl, 1.2 KH_2_PO_4_, 129 NaCl, 5 KaHCO_3_, 1.2 MgSO_4_, 1.8 CaCl_2_, and 10 HEPES with pH adjusted at 7.4 using NaOH supplemented with 5.6 mM glucose. Isolated single neurons were cultured for 12∼36 h in Eagle’s minimal essential medium containing 5.6 mM glucose supplemented with 10% feral bovine serum, 100 µg/mL streptomycin, and 100 units/mL penicillin.

### Measurements of [Ca^2+^]_i_ in NG Neurons

Measurements of [Ca^2+^]_i_ in primary cultured NG neurons were carried out by fura-2 fluorescence imaging as reported previously [14]. Briefly, following incubating with 2 µM fura-2 AM (DOJINDO, Kumamoto, Japan) for 30 min at 37°C, the cells were mounted in a chamber and superfused with HKRB containing 5.6 mM glucose at 1.3 ml/min at 30°C. Fluorescence ratio images at 510 nm due to excitation at 340 and 380 nm were produced by an Aquacosmos ver. 2.5 (Hamamatsu Photonics, Shizuoka, Japan). NG neurons were selected by their round shape, while non-neuronal cells had spindle or filamentous shape. When [Ca^2+^]_i_ changed within 5 min after addition of agents and their amplitudes were at least twice larger than the spontaneous fluctuations of the baseline, they were considered responses. Data were taken exclusively from the neurons that responded to 55 mM KCl. In all experiments, neurons from at least three separate preparations were used to ensure that the observed responses were representative.

### Patch-clamp Experiments in NG Neurons

Perforated whole-cell currents were recorded using a pipette solution containing amphotericin B (150 µg/mL, Sigma) dissolved in 0.1% DMSO as previously described [15]. Membrane potentials were recorded using an amplifier (Axopatch 200B; Molecular Devices, Foster, CA) in a computer using pCLAMP 9.2 software. HKRB containing 5.6 mM glucose was used as the bath solution. Pipette solution contained (in mM) K_2_SO_4_ 40, KCl 50, MgCl_2_ 5, EGTA 0.5, HEPES 10 at pH 7.2 with KOH. We used electrode resistance of 3∼5 MΩ. NG neurons derived from ICR mice were voltage-clamped at a holding potential of -70 mV after successful establishment of perforated whole-cell clamp mode with series resistance less than 20 MΩ, followed by switching to current clamp mode to record membrane potential. The electrophysiological experiments were performed at room temperature (RT, 25°C).

### Immunocytochemistry and Identification of CART-containing NG Neurons

After [Ca^2+^]_i_ measurements, the cells were fixed with 4% paraformaldehyde for 2 hr at RT, and processed for CART immunoreactivity as described previously [16]. Anti-CART (55–102) antibody (H-003-62, 1∶10,000, Phoenix pharmaceuticals, CA) was used. The neurons in which [Ca^2+^]_i_ was recorded were correlated with their corresponding immunocytochemical results based on the phase-contrast photographs of the neurons taken right after [Ca^2+^]_i_ measurements and the photographs of the neurons after immunostaining, as described previously [16].

### Labeling NG Neurons that Innervate Pancreas Using Retrograde Tracer

The specific subpopulation of NG neurons that innervate pancreas were labeled with retrograde tracer, 1,1′-dioctadecyl-3,3,3′,3′-tetramethylindocarbocyanine perchlorate (DiI; DiIC18(3)) (Life technologies, CA). DiI solution (3 mg/mL, 15% DMSO in sterile saline) was injected into 20–30 sites (1 µL/site) of the overall pancreas using 30-G needle connected to a microsyringe (Hamilton, NV) in ICR mice anesthetized with tribromoethanol (200 mg/kg, ip). The injection area was swabbed to remove any excess of tracer, the wound was sutured shut, and the mice were allowed to recover. Three weeks after injection, NG neurons were dissected out of the mice and cultured as mentioned above. DiI-labeled neurons were identified by the fluorescence with 549 nm excitation and subjected to [Ca^2+^]_i_ measurements.

### Reverse Transcriptase (RT)-PCR

Total RNA of NGs in ICR mouse was isolated using TRIzol (Life technologies) and treated with RQ1-DNase (Promega, WI) to remove residual contaminations of DNA. The first-strand cDNA synthesis was completed using the ReverTra Ace (TOYOBO, Osaka, Japan). PCR were examined by MightyAmp DNA Polymerase (TaKaRa Bio, Shiga, Japan) (94°C for 10 sec, 60°C for 15 sec, and 68°C for 20 sec × 25 to 35 cycles) and agarose gel electrophoresis for correct product size. Primers sequence and product length were as follows: IR sense, 5′-ATGGGCTTCGGGAGAGGAT-3′, antisense, 5′-GGATGTCCATACCAGGGCAC-3′, 121 bp; IRS-2 sense, 5′-CTACCCACAGAGCCCAAGAG-3′, antisense, 5′-CCAGGGATGAAGCAGGACTA-3′, 151 bp.

### Tissue Preparation and Immunohistochemistry for IR

ICR mice deeply anesthetized with urethane (1.5 g/kg, ip) were transcardially perfused with saline containing heparin (20 IU/mL) and then 4% paraformaldehyde in 0.1 M phosphate buffer. Isolated nodose ganglia were immediately postfixed in the same fixative for 2 hr at 4°C, and the pancreases for overnight. After post-fixation, they were immersed in PBS containing 25∼30% sucrose for 1∼2 days at 4°C, embedded in O.C.T. Compound (Sakura Finetek Japan, Tokyo, Japan), frozen and cut into slice sections (8 µm for NG, 20 µm for pancreas) using a precision cryostat (Leica Microsystems, IL). The sections were treated with blocking solution (2% normal goat serum and 2% BSA in PBS) for 30 min at RT, and then incubated with a rabbit polyclonal antibody against insulin receptor β-subunit (IR-β, sc-711, 1∶200, Santa cruz biotechnology, CA) or with a mouse monoclonal antibody against neurofilament (M0769, 1∶500, Dako, Denmark) for overnight at 4°C. After the sections were rinsed with PBS, they were incubated with secondary antibody (Alexa 488 goat anti-rabbit IgG or Alexa 594 goat anti-mouse IgG, 1∶500, Life technologies) for 30 min at RT. Control experiments were carried out without the primary antibody. Fluorescence images were acquired with a BX50 microscope and a DP50 digital camera (Olympus, Tokyo, Japan).

### Measurements of Insulin Concentration in Pancreatic Artery, Pancreatic Vein, and Portal Vein in Rats

Male Wistar rats after overnight fasting or with *ad lib* condition were used. The blood samplings were performed at 10∶00∼11∶00, as previously reported [17]. Briefly, after injection of pentobarbital anesthesia (50 mg/kg, ip) to the rats, blood samples were collected from the pancreatic artery (celiac artery) and vein (splenic vein) and portal vein. Plasma insulin concentrations were measured using an enzyme-linked immunosorbent assay (ELISA) kit (Morinaga Institute of Biological Science, Yokohama, Japan).

### Measurements of AKT Phosphorylation in Nodose Ganglia after Endogeneous Insulin Secretion by Glibenclamide Injection

ICR mice fasted for 4 hr were acutely ip injected with glibenclamide (1.5 mg/kg, 10 mL/kg constructed by 0.1% DMSO, 2% Tween 80, and 97.9% saline) at 13∶00. Blood samples were collected from the tail vein at 0, 10, and 20 min after ip injection. Insulin concentrations in each plasma samples were measured using an ELISA kit (Morinaga Institute of Biological Science). After blood sampling at 20 min, NGs were isolated from the mice under isoflurane anesthesia, washed with ice-cold PBS, and acutely frozen until western bloods analysis. A sample was composed of a right and a left NG derived from a mouse. NG cells were lysed in 50 µL lysis buffer (100 mM NaCl, 0.5% NP40, 1 mM EDTA, 10 mM Tris–HCl with pH 7.5, 100 mM sodium fluoride, 10 mM sodium PPi, 2 mM sodium orthovanadate, and 1 mM PMSF). The 10 µg protein were subjected to 10% SDS–PAGE and transferred to nitrocellulose filters. AKT and phospho-Ser473 AKT were detected with the polyclonal antibody (1∶1,000, Cell Signaling Technology). Immunoreactive proteins were detected with HRP-conjugated secondary antibody and the ECL system (GE healthcare, Buckinghamshire, UK). Immunoreactive signal was quantified by using LAS-1000 (FUJIFILM, Tokyo, Japan).

### Preparation of Single Neurons of Hypothalamic Arcuate Nucleus and Measurement of [Ca^2+^]_i_

The arcuate nucleus (ARC) was excised from the brain of C57BL/6J and IRS2-KO mice aged 2∼4 months, and then, single neurons were prepared, as previously reported [16], [18]. Briefly, the whole ARC was dissected out and incubated with HKRB containing 20 units/ml papain (Sigma), 1 mM cystein (Sigma), 0.015 mg/ml deoxyribonuclease (Sigma), 0.75 mg/ml BSA (Sigma) and 10 mM glucose for 17 min at 36°C, followed by gentle mechanical trituration for 5∼10 min. The single neurons obtained were distributed onto coverslips and incubated in the humidified chamber at 30°C for 30 min to 6 hr until use.

Measurements of [Ca^2+^]_i_ in ARC neurons were carried out by ratiometric fura-2 fluorescence imaging with 510 nm emission and 340/380 nm excitation by Argus-50 system (Hamamatsu Photonics, Shizuoka, Japan) as reported previously [16], [18]. HKRB containing 10 mM glucose was used as the perfusate.

### Data Analysis

All data were shown as means ± SEM. Statistical analysis was performed by one-way ANOVA followed by Tukey’s or Dunnett’s multiple comparison tests, unpaired t-test, or chi-square test using the Prism 4 (GraphPad Software, CA). *P*<0.05 was considered significant.

## Results

### Insulin Concentration-dependently Increases [Ca^2+^]_i_ in NG Neurons

**Figure 1:**
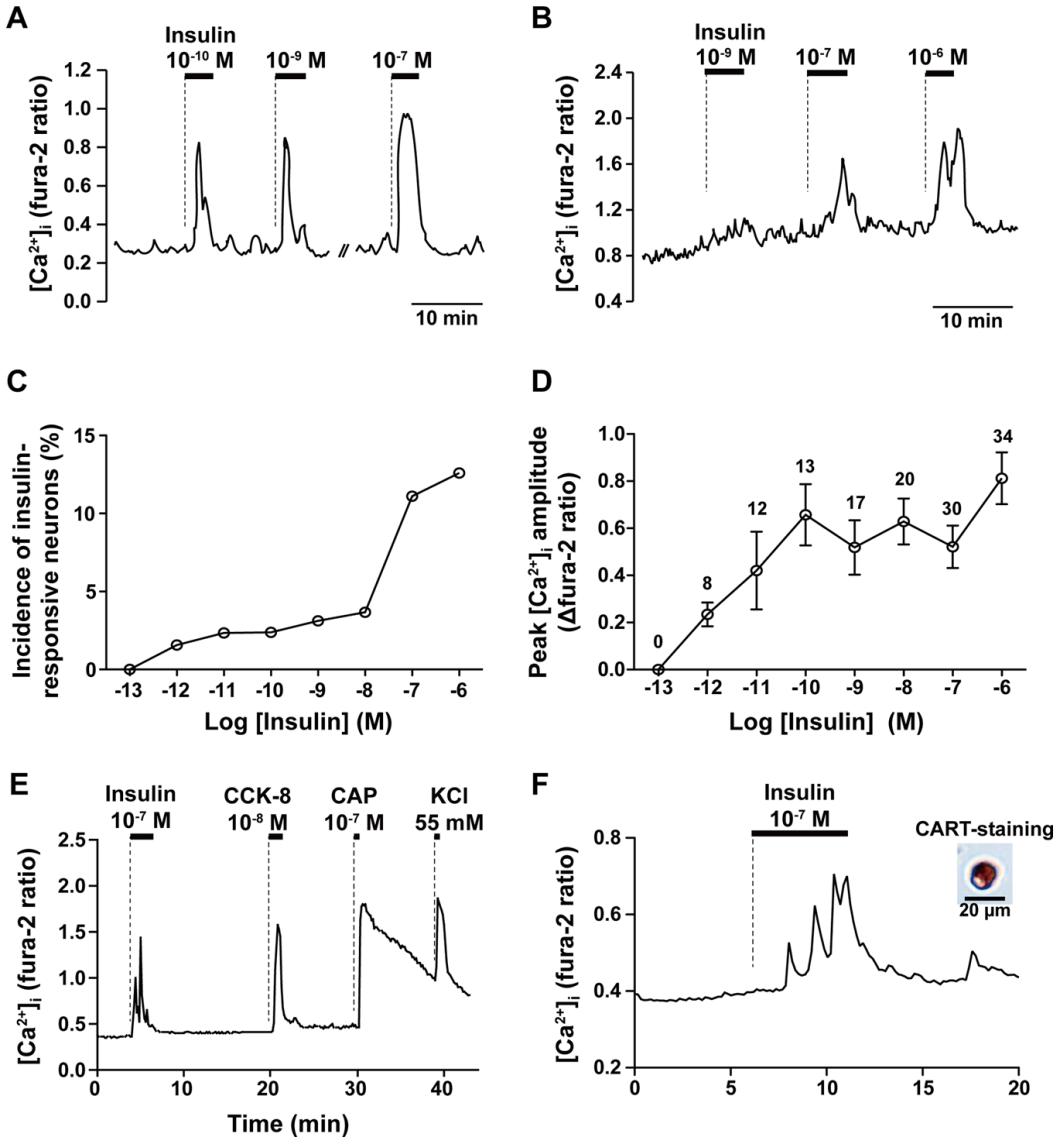
Insulin increases [Ca^2+^]_i_ in NG neurons isolated from mouse vagal afferents that responded to CCK-8 and CAP and contained the neuropeptide CART. ***A and B***: Repetitive administration of increasing concentrations of insulin at 10^−13^∼10^−6^ M induced repeated [Ca^2+^]_i_ increases in a concentration-dependent manner in a single NG neuron. [Ca^2+^]_i_ is expressed by fura-2 ratio and bars above the tracings indicate the periods of administration of agents specified. The [Ca^2+^]_i_ recordings are representative of the NG neurons that responded to insulin at 10^−10^, 10^−9^ and 10^−7^ M (n = 9, *A*), and that responded to insulin at 10^−7^ and 10^−6^ M but not at 10^−9^ M (n = 15, *B*). ***C and D***: Concentration-dependent effects of insulin to increase [Ca^2+^]_i_ in single NG neurons. Incidence of [Ca^2+^]_i_ responses is expressed by the percentage of neurons that responded to insulin (*C*). The numbers of the NG neuron that responded to insulin over those to 55 mM KCl are 0/511 for insulin at 10^−13^ M, 8/511 at 10^−12^ M, 12/511 at 10^−11^ M, 13/546 at 10^−10^ M, 17/546 at 10^−9^ M, 20/546 at 10^−8^ M, 30/270 at 10^−7^ M and 34/270 at 10^−6^ M. Amplitudes of [Ca^2+^]_i_ responses to insulin in insulin-responsive neurons are expressed by increases in fura-2 fluorescence ratio (*D*). The numbers above each point indicate the number of the neuron that responded to insulin. *E:* The result is representative of 37 neurons that responded to insulin at 10^−7^ M, CCK-8 at 10^−8^ M and CAP at 10^−7^ M. Out of 53 neurons that responded to insulin, 37 (70%) neurons responded to CCK-8 and all responded to CAP. *F:* Insulin at 10^−7^ M increased [Ca^2+^]_i_ in a NG neuron that was subsequently proved to be immunoreactive to CART. Out of 23 NG neurons that responded to insulin, 13 (56%) neurons were immunoreactive to CART.

We measured direct effect of insulin on [Ca^2+^]_i_ in the single NG neurons isolated from ICR mice. Insulin at 10**^−^** ^13^∼10^−6^ M was sequentially applied to NG neurons with 4 min pulses and at least 8 min washing periods. As shown in Fig. 1A and B, a NG neuron responded to insulin at 10^−10^, 10^−9^ and 10^−7^ M, while another NG neuron responded to insulin at 10^−7^ and 10^−6^ M. Insulin at 10^−12^∼10^−6^ M, but not 10^−13^ M, increased [Ca^2+^]_i_ (Fig. 1C and D). The incidence (Fig. 1C) and amplitude (Fig. 1D) of [Ca^2+^]_i_ responses took plateau with insulin at 10^−11^∼10^−8^ M, and the incidence further increased at 10^−7^ M and 10^−6^ M insulin, thus showing a concentration-dependency with two components (Fig. 1C).

### Relationship between Insulin-, CCK- and CAP-responsive NG Neurons

CCK-8 and capsaicin (CAP) are the well characterized substances that directly activate NG neurons. We examined whether insulin-responsive neurons are distinct from or overlap with CCK-8- and/or CAP-responsive neurons in the NG. NG neurons were exposed sequentially to insulin (10^−7^ M), CCK-8 (10^−8^ M), CAP (10^−7^ M), and KCl (55 mM) (Fig. 1E). We previously reported that 10^−8^ M for CCK-8 and 10^−7^ M for CAP are the maximal doses for inducing [Ca^2+^]_i_ increases in NG neurons [14], [19]. Insulin, CCK-8, and CAP raised [Ca^2+^]_i_ in 53 of 500 (10.6%), 207 of 500 (41.4%), and 307 of 500 (61.4%) neurons examined, respectively. Among 53 NG neurons that responded to insulin, 37 neurons (70%) responded to CCK-8 and all to CAP. Thus, the insulin responders were largely CCK responders and entirely CAP responders.

### Neurochemical Characterization of Insulin-responsive Neurons

The NG neurons highly express CART [20]. We examined whether insulin-responsive NG neurons contain CART. The effects of insulin on [Ca^2+^]_i_ were measured in single NG neurons, which were subsequently immunostained with antibody against CART. The neuron exemplified in Fig. 1F responded to insulin with an increase in [Ca^2+^]_i_ and was subsequently proved to be immunoreactive to CART. Out of 23 neurons that responded to insulin, 13 were shown to be immunoreactive to CART (56.5%). This incidence was significantly higher than the incidence of the CART- immunoreactive neurons in the total NG neurons (61/166, 36.7%), indicating that insulin preferentially targets CART neurons.

### Insulin Induces Depolarization and Voltage-gated Ca^2+^ Influx in NG Neurons

**Figure 2:**
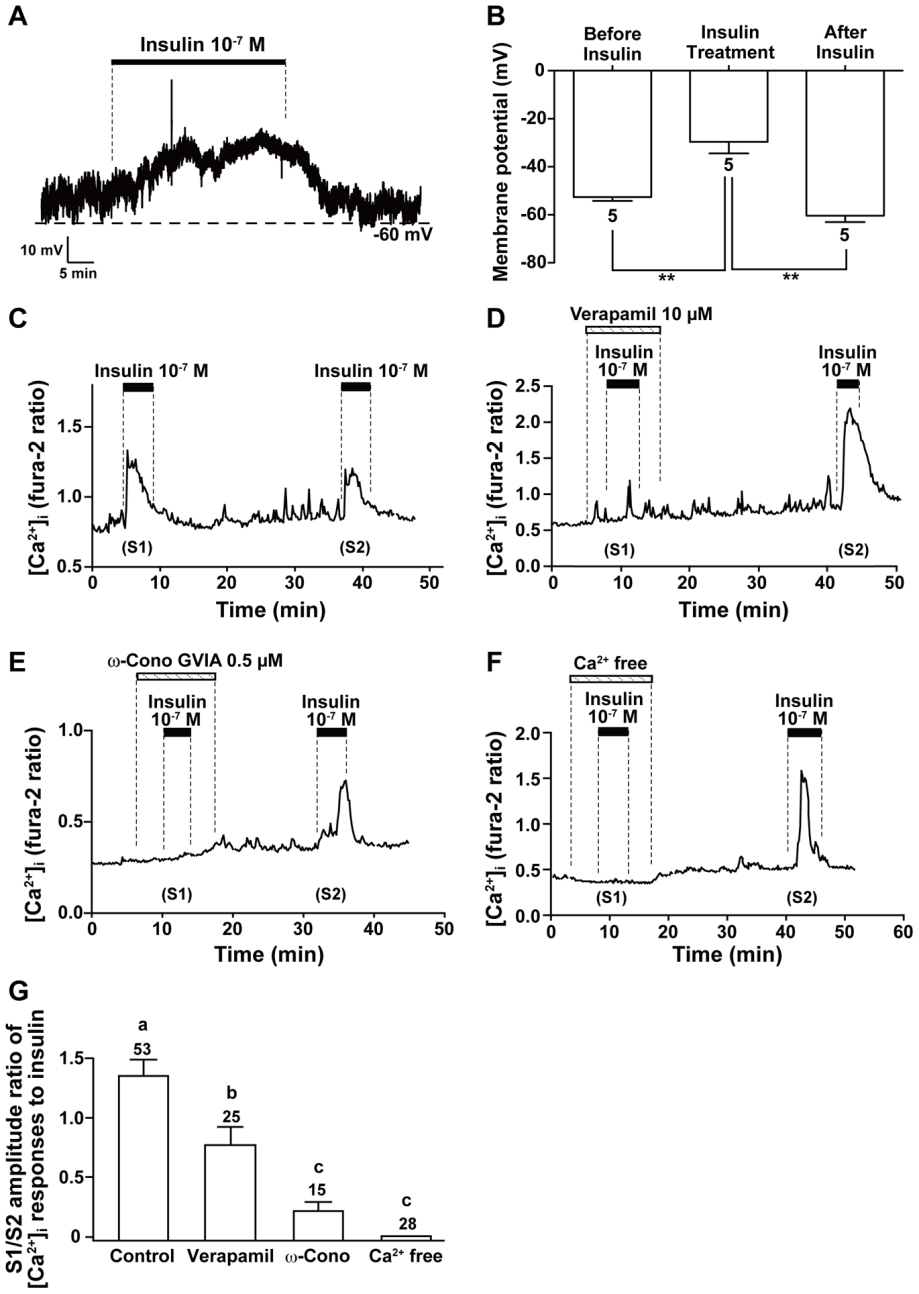
Insulin depolarizes plasma membrane and induces voltage-gated Ca^2+^ influx in NG neurons. *****A and B*****: Administration of 10^−7^ M insulin depolarized the membrane potential in NG neurons under perforated-patch configuration (*A*). The results are representative of 5 neurons. The membrane potentials before, during and after insulin treatment (*B*, n = 5) ** p<0.01 by one-way ANOVA followed by Turkey’s test. ***C***: Administration of 10^−7^ M insulin twice induced repeated [Ca^2+^]_i_ increases in single NG neurons. The data are representative of 53 neurons. ***D∼F***: Insulin-induced [Ca^2+^]_i_ increases were markedly suppressed by L- and N-type Ca^2+^ channel blockers, 10 µM verapamil (*D*) and 0.5 µM ω-conotoxin GVIA (ω-Cono; *E*), respectively, and abolished under a Ca^2+^-free condition added with 0.1 mM EGTA (*F*). The data are representative of 25 neurons in (*D*), 15 in (*E*) and 28 in (*F*). ***G***: The ratio of the peak [Ca^2+^]_i_ response to the first insulin stimulation (S1) over that to the second insulin stimulation (S2) under control and test conditions. The numbers on each bar indicate the numbers of neurons that responded to insulin. Different letters above bars indicate significant difference, *P*<0.05 by one-way ANOVA followed by Tukey’s test.

The resting membrane potential of single NG neurons was -52.7±1.9 mV under the perforated-patch configuration. As sown in Fig. 2A and B, administration of 10^−7^ M insulin depolarized membrane potential in 5 of 55 NG neurons (9.1%) in a reversible manner (−29.7±4.8 mV with insulin vs. −60.4±2.7 mV after washout of insulin). In [Ca^2+^]_i_ measurements, repeated administration of 10^−7^ M insulin twice with a washing period of 20∼30 min increased [Ca^2+^]_i_ repetitively in NG neurons (Fig. 2C). The amplitude of [Ca^2+^]_i_ response to the first stimulation (S1) was somewhat larger than that to the second stimulation (S2) (Fig. 2C and G). The effects of verapamil and ω-conotoxin GIVA, the voltage-gated L- and N-type Ca^2+^ channel blockers, respectively, were examined for the [Ca^2+^]_i_ responses to S1. The insulin (10^−7^ M)-induced increase in [Ca^2+^]_i_ was markedly suppressed in the presence of verapamil (10 µM) (Fig. 2D) and ω-conotoxin GIVA (0.5 µM) (Fig. 2E) compared to the [Ca^2+^]_i_ responses to insulin observed after washing out the agents. ω-Conotoxin GIVA showed a greater inhibition than verapamil (Fig. 2G). In a Ca^2+^-free condition made with no added Ca^2+^ and 0.1 mM EGTA, insulin administration failed to increase [Ca^2+^]_i_ in all the neurons examined, and after bringing Ca^2+^ back to HKRB it elicited [Ca^2+^]_i_ increases (Fig. 2F and G).

### Expression of IRs on NG Neurons

**Figure 3:**
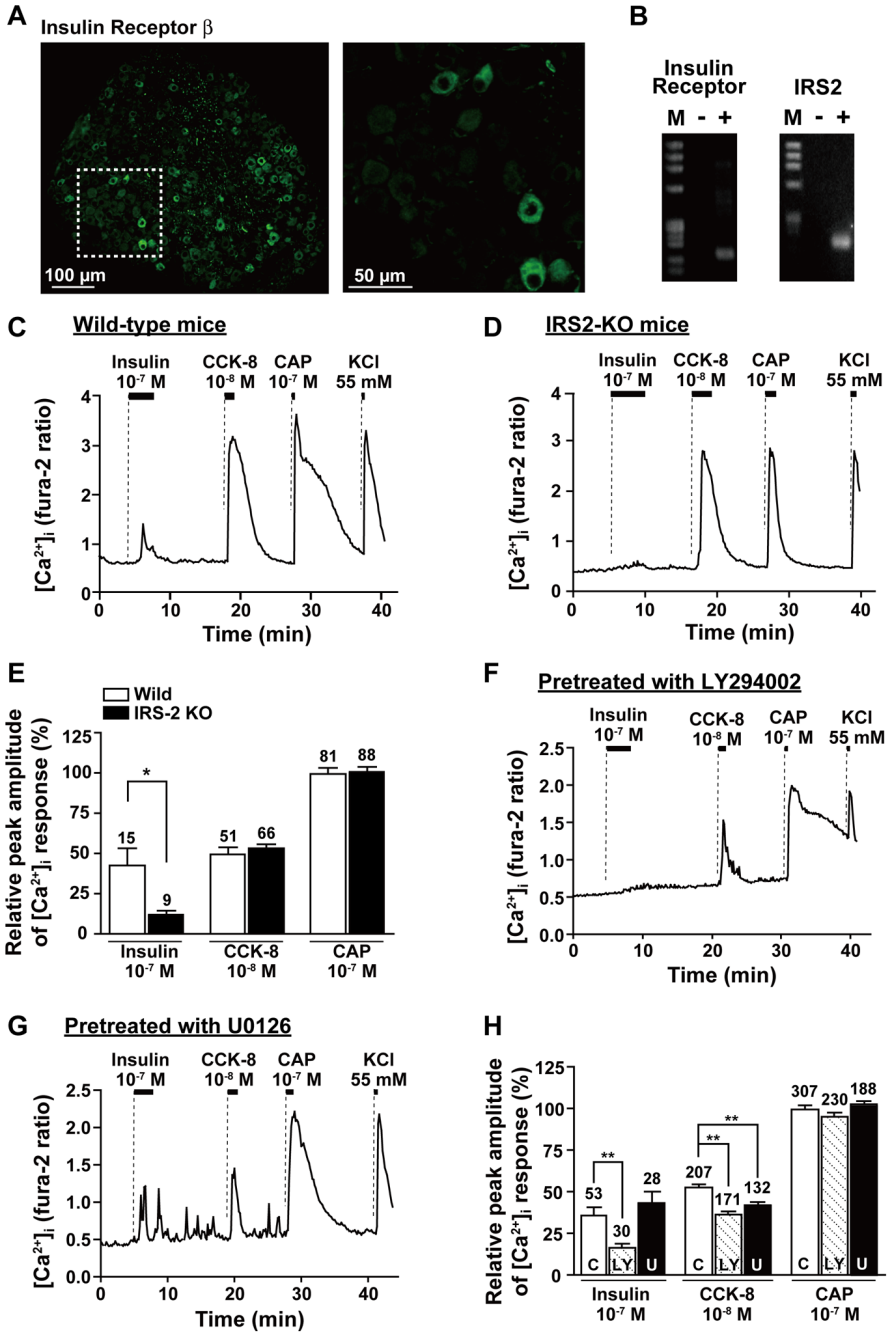
IRs and IRS2 are expressed in NG neurons, and inhibition of IRS2 and PI3K but not MAPK suppresses insulin-induced [Ca^2+^]_i_ increases in NG neurons. ***A***: Neurons immunoreactive to IR-β, revealed by Alexa 488 fluorescence, were observed in the section of mouse NG. The picture is representative of 13 sections from 4 mice. The right picture is the magnification of the area marked with dotted line in the left picture. ***B***: mRNA expressions for IRs and IRS2 in NGs. Representative electrophoretic patterns of RT-PCR products of IR (left) and IRS2 (right) mRNAs in NGs. M = size maker, (−) = RT (−) as a negative control, (+) = RT (+). ***C∼E***: Effects of insulin (10^−7^ M), CCK (10^−8^ M), and CAP (10^−7^ M) on [Ca^2+^]_i_ in NG neurons derived from wild-type C57BL/6J (*C*) and IRS2 knockout mice (*D*). The [Ca^2+^]_i_ response to insulin, but not CCK-8 and CAP, were markedly attenuated in the NG neurons of IRS2 knockout mice (*D*, n = 9) as compared with wild type (*C*, n = 14). The amplitude of [Ca^2+^]_i_ response to each regent is normalized to that to KCl in each neuron and expressed as relative values (percentage) (*E*). The numbers on each bar indicate the numbers of neurons that responded to insulin, CCK-8 or CAP. The amplitude of [Ca^2+^]_i_ responses to insulin were suppressed by IRS2-deficiency. ***F∼H***: Effects of a PI3K inhibitor LY294002 at 50 µM (LY, pretreatment for 1 hr, *F*) and a MAPK inhibitor U0126 at 10 µM (U, pretreatment for 0.5 hr, *G*) on insulin-induced [Ca^2+^]_i_ increases in NG neurons. The [Ca^2+^]_i_ traces of control experiments (C) are shown in Fig. 1E. In these three conditions, the amplitude of [Ca^2+^]_i_ response to each regent is normalized to that to KCl in each neuron and expressed as relative values (percentage) (*H*). The numbers on each bar indicate the numbers of neurons that responded insulin, CCK-8 or CAP. [Ca^2+^]_i_ responses to insulin were suppressed by LY but not U. *P<0.05, **P<0.01 by paired t-test (*E*) and one-way ANOVA followed by Dunnett’s test vs. control (*H*).

Immunohistochemistry using antibody against IR revealed an intense immunoreactivity in the cell membrane and cytoplasm of NG neurons. A substantial fraction of NG neurons (13.4±1.2%, n = 13 including 6 right and 7 left NG sections) were immunoreactive to IR (Fig. 3A). No difference was observed in the expression of IR between right and left nodose ganglia. IR mRNA was expressed in NGs (Fig. 3B).

### Insulin-induced [Ca^2+^]_i_ Increases Involve IRS2 and PI3K, but not MAPK, in NG Neurons

We next examined whether IRS2, PI3K and/or mitogen-activated protein kinase (MAPK), key molecules for insulin signaling, are involved in the insulin-induced [Ca^2+^]_i_ increases in NG neurons. IRS2 mRNA expression was detected in NGs using RT-PCR analysis (Fig. 3B). Hence, IRS2-KO mice were investigated. Compared to wild-type mice, the male IRS2-KO mice aged 3 months, used in this study, exhibited increased body weight (28.0±0.6 g for wild type vs. 34.2±0.7 g for KO) and blood glucose (122.5±6.4 mg/dL for wild type vs. 141.0±5.2 mg/dL for KO, at 19∶00). Insulin at 10^−7^ M induced marked increases in [Ca^2+^]_i_ in NG neurons isolated from wild type mice and cultured overnight (Fig. 3C), while it induced only small increases in [Ca^2+^]_i_, if any, in the neurons from IRS2-KO mice (Fig. 3D). The incidence of [Ca^2+^]_i_ responses to insulin tended to decrease in NG neurons of IRS2-KO mice (9/134, 6.7%) compared to wild-type mice (15/128, 11.7%), and the amplitude of [Ca^2+^]_i_ responses to insulin were significantly smaller in NG neurons of IRS2-KO than wild type mice (Fig. 3E). On the other hand, administration of CCK-8 (10^−8^ M) and CAP (10^−7^ M) increased [Ca^2+^]_i_ in NG neurons derived from wild-type and IRS2-KO mice in a similar manner (Fig. 3C∼E).

Preincubation for 1 hr with 50 µM LY294002, a PI3K inhibitor, significantly attenuated the amplitude of [Ca^2+^]_i_ responses to insulin in NG neurons compared to control (Fig. 3F and H vs. Fig. 1E), whereas preincubation with 10 µM U0126, a MAPK inhibitor, for 30 min had no effect (Fig. 3G and H vs. Fig. 1E). Additionally, pretreatment with LY293002 and U0126 slightly decreased the amplitude of [Ca^2+^]_i_ responses to CCK-8, but not that to CAP (Fig. 3H). These results indicate that IRS2 and PI3K are involved in the insulin-induced [Ca^2+^]_i_ increases.

### Insulin Concentration is High in the Pancreas, NG Neurons Innervate the Pancreas, and Insulin Activates the Pancreas-innervating NG Neurons with Greater Incidence

A much greater population of NG neurons were recruited to the [Ca^2+^]_i_ response to insulin when its concentration was elevated to 10^−7^ M (Fig. 1C), a concentration 100∼1,000-fold higher than that in circulation. We thought that this result might reflect a situation in which insulin released from pancreatic islets at high concentrations could be immediately sensed by the NG neurons that innervate the pancreas. To verify this, we examined 1) whether insulin concentration in/around the pancreas is actually high, 2) whether neuronal fibers innervating the pancreatic islet express IR, and 3) whether the NG neurons innervating the pancreas could sense insulin particularly at higher concentrations.

**Figure 4:**
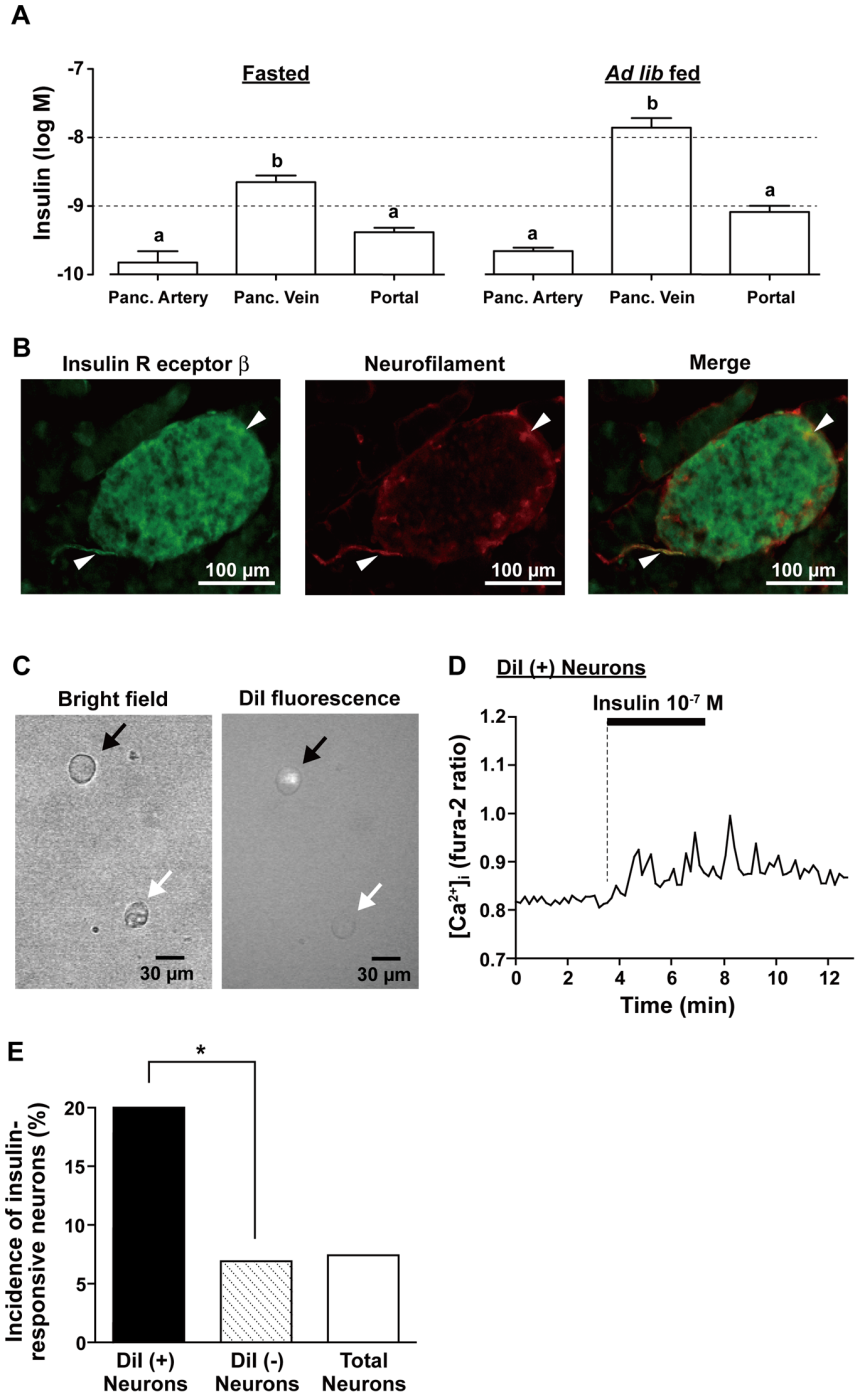
Insulin concentration is high in pancreas, NG neurons innervate pancreas, and insulin activates pancreas-innervating NG neurons with greater incidence. ***A*** *:* Insulin concentrations in pancreatic vein (Panc. Vein) were higher than those in pancreatic artery (Panc. Artery) and portal vein (Portal) in fasted (left, n = 6) and *ad lib* fed rats (right, n = 10). Different letters above bars indicate significant difference, *P*<0.05 by one-way ANOVA followed by Tukey’s test. ***B***: Immunofluorescence micrographs for IR-β (left; green), neurofilament (center; red), and both (right; merged). Immunoreactivity to IR-β was localized in the fibers/terminals of neurons that innervate pancreatic islets. Arrowheads show the neurons immunoreactive to both neurofilament and IR-β. ***C***: Bright field (left) and DiI fluorescence (right) microphotographs indicate isolated NG neurons labeled (black arrow) and unlabeled (white arrow) with DiI injected into pancreas. ***D***: A neuron pointed by black arrow in (*C*) responded to insulin at 10^−7^ M with [Ca^2+^]_i_ increases. ***E***: In total 625 neurons including labeled and unlabeled neurons, 46 neurons (7.4%) responded to insulin with [Ca^2+^]_i_ increases. DiI-labeled neurons responded to insulin with [Ca^2+^]_i_ increases with a much greater incidence (filled bar, 5 of 25 cells, 20.0%) than unlabeled neurons (dotted bar, 41 of 600 cells, 6.8%). *P<0.05 by chi-square test.

First, to assess local insulin concentrations in the pancreas, we measured the insulin concentration in the pancreatic vein. Furthermore, to estimate the difference between the insulin concentration in the pancreas and that in circulation, we compared insulin concentration in the pancreatic vein, that in the pancreatic artery and that in the portal vein. Under the condition fasted overnight, insulin concentration in the pancreatic vein (2.3±0.57 nM) was 15-fold higher than that in the pancreatic artery (0.15±0.069 nM) and 5-fold higher than that in the portal vein (0.41±0.066 nM) (Fig. 4A left). In *ad lib* fed condition, insulin concentration in the pancreatic vein (14±5.3 nM) was 64-fold higher than that in the pancreatic artery (0.22±0.027 nM) and 17-fold higher than that in the portal vein (0.82±0.19 nM) (Fig. 4A right). Moreover, insulin concentration in the pancreatic vein under *ad lib* fed condition (not right after meal) was greater than that under fasting condition and reached a level higher than 10^−8^ M (Fig. 4A right).

Second, we investigated immunohistochemically whether the neurons innervating pancreatic islets express IRs. Neural fibers were detected in and around the islets using anti-neurofilament antibody (Fig. 4B, center; red), and some of neuronal fibers that innervated pancreatic islets were shown to express IRs (Fig. 4B, arrowhead).

Third, we examined whether insulin interacts with the NG neurons that innervate pancreas. These neurons were labeled with retrograde tracer DiI injected into the pancreas. Isolated NG neurons that had innervated the pancreas were identified by the DiI fluorescence under the fluorescent microscope before [Ca^2+^]_i_ measurements (Fig. 4C), and 25 (4.0%) of 625 KCl-responsive neurons were DiI fluorescence-positive. Among these 25 DiI-positive neurons, 5 neurons (20.0%) responded to 10^−7^ M insulin (Fig. 4D and E). On the other hand, 41 of 600 DiI-unlabeled neurons (6.8%) responded to 10^−7^ M insulin (Fig. 4E). Thus, the insulin response took place in DiI-labeled neurons at a significant higher rate than in unlabeled neurons (Fig. 4E), suggesting that vagal afferent neurons innervating to the pancreas respond to insulin with greater frequency.

### Glibenclamide Injection Induced Insulin Release and AKT Phosphorylation in NG Neurons

**Figure 5:**
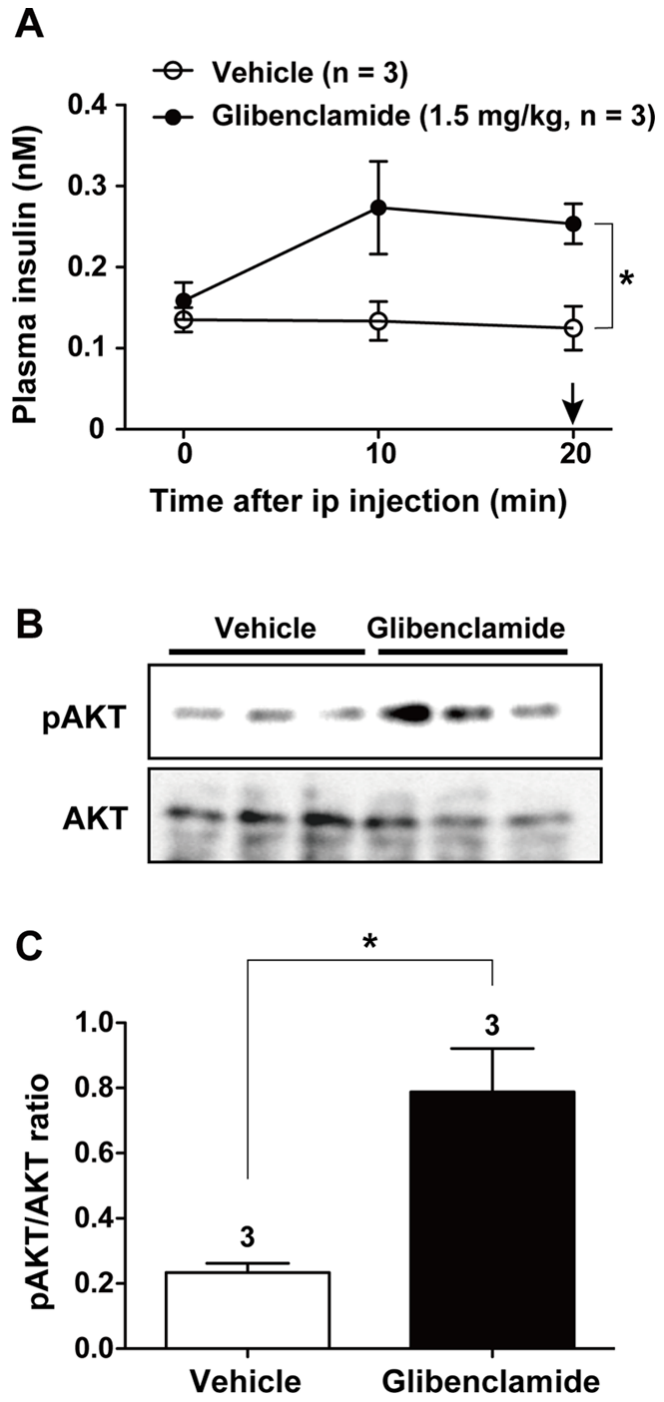
Injection of glibenclamide induces insulin release and AKT phosphorylation in NG neurons. *****A*****: Plasma insulin levels were determined after ip injection of vehicle or glibenclamide (1.5 mg/kg). Arrow at 20 min indicates the time for isolation of NGs. ***B and C***: Levels of AKT phosphorylation in NGs of mice at 20 min after injection. The intensity of AKT phosphorylation is expressed by the ratio of phosphorylated over total AKT proteins (*C*). In C, the number on each bar indicates sample numbers. *P<0.05 by unpaired t-test.

We examined whether insulin released by administration of sulfonylurea glibenclamide could be sensed by NG neurons. Effect of insulin on NG neurons was assayed by phosphorylation of AKT, the principal kinase downstream of the insulin/IR/IRS2/PI3K signaling. Intraperitoneal injection of glibenclamide (1.5 mg/kg) evoked insulin secretion at 10 and 20 min after injection (Fig. 5A). Right after taking blood sample for insulin assay, tissue was dissected out from mice and subjected to measurement of phosphorylation. At 20 min after glibenclamide injection, the phosphorylation of AKT in NG neurons was elevated (Fig. 5B and C). These results suggested that insulin secreted by glibenclamide might activate NG neurons.

### Insulin Suppresses Ghrelin-induced [Ca^2+^]_i_ Increases in ARC Neurons from IRS2 Knockout Mice

**Figure 6:**
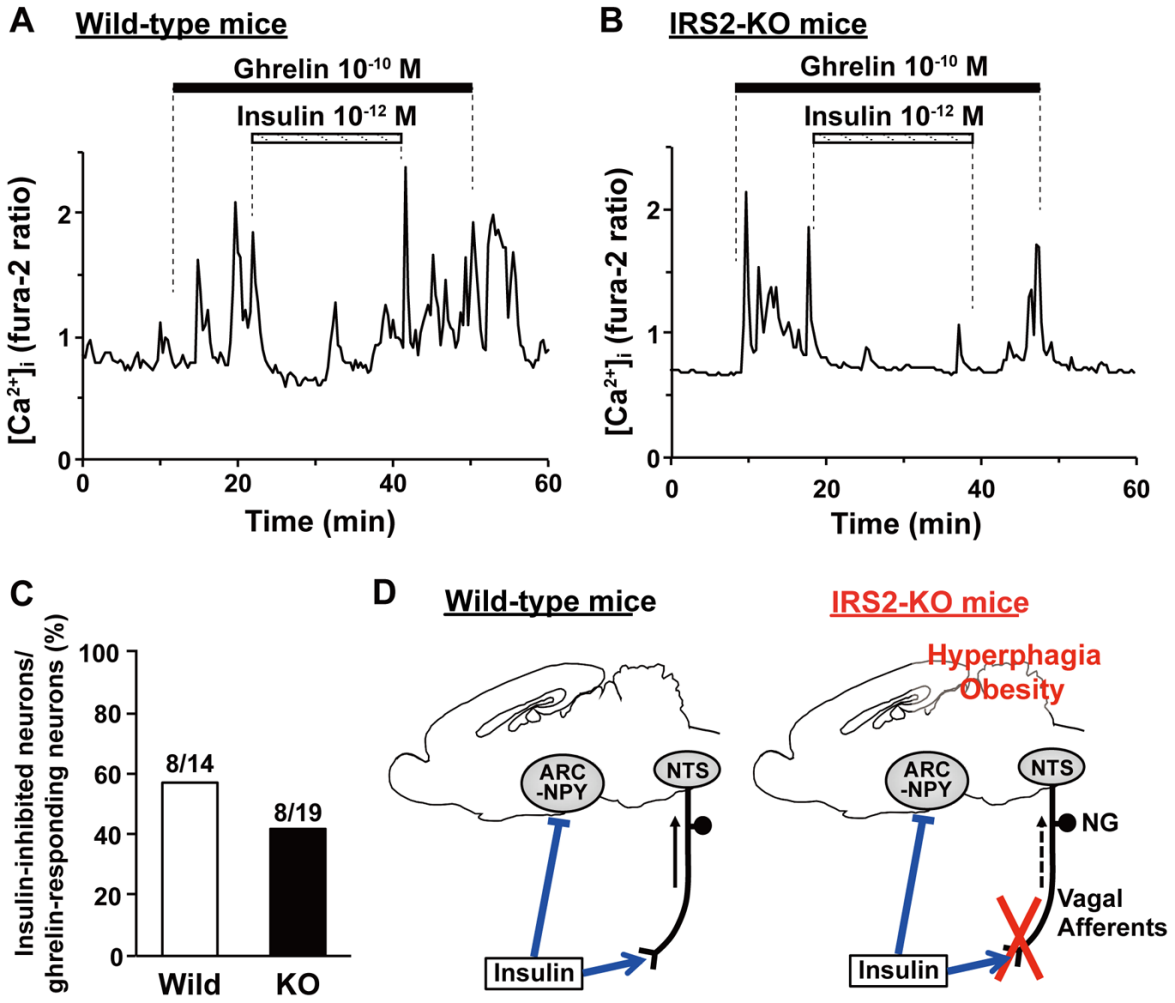
Insulin suppresses ghrelin-induced [Ca^2+^]i increases in ARC neurons from IRS2 knockout mice. *****A and B*****: Insulin suppressed ghrelin-induced [Ca^2+^]_i_ increases in ARC neurons from wild type mice (*A*, n = 8) and IRS2-KO mice (*B*, n = 8) in a similar manner. ***C***: The percentage of insulin-inhibited neurons among ghrelin-activated ARC neurons. The numbers above each bar indicate the number of insulin-inhibited neurons over that of ghrelin-responsive neurons. ***D***: Hypothetical model for the role of IRS2-dependent insulin reception by vagal afferent NG neurons. In IRS2-KO mice, insulin action to activate nodose ganglion (NG) neurons is severely impaired (Fig. 3C∼E) while insulin action to inhibit first order orexigenic ghrelin-responsive NPY neurons in ARC [18] is intact (Fig. 6A∼C). Hence, hyperphagic obese phenotype parallels with impaired insulin action on NG neurons in IRS2-KO mice. Therefore, the IRS2-dependent reception of insulin by NG neurons could be implicated in the regulation feeding and body weight.

In addition to the action on NG neuron, insulin is known to pass through BBB and directly act on the first order neurons in the ARC of hypothalamus. It is known that insulin inhibits orexigenic neuropeptide Y (NPY)/agouti-related protein (AgRP) neurons, as well as activating proopiomelanocortin (POMC) neurons, in ARC, and that this action contributes to the anorexigenic effect of insulin. Insulin was shown to suppress ghrelin-induced [Ca^2+^]_i_ increase in ARC NPY/AgRP neurons [18]. In this study we examined whether this insulin action is impaired in IRS2-KO mice. As depicted in Fig. 6, administration of 10^−10^ M ghrelin increased [Ca^2+^]_i_ in ARC neurons and the ghrelin-induced [Ca^2+^]_i_ increase was suppressed by 10^−12^ M insulin in single ARC neurons isolated from IRS2-KO mice (Fig. 6B), and the magnitude of suppression was indistinguishable from that observed in ARC neurons from wild-type mice (Fig. 6A). Insulin exerted this inhibitory effect on approximately half of the ghrelin-responsive ARC neuron in both wild-type and IRS2-KO mice (Fig. 6C). Thus, insulin action to inhibit orexigenic ARC neurons was unaltered in IRS2-KO mice as compared to wild-type mice. Taken together, in IRS2-KO mice, insulin action on the first order hypothalamic orexigenic neurons was intact (Fig. 6) whereas insulin action on NG neuron was markedly reduced (Fig. 3C∼E). Thus, hyperphagia and obesity observed in IRS2-KO mice was paralleled with the impaired sensing of insulin by NG neuron (Fig. 6D).

## Discussion

The present study, for the first time, revealed that insulin directly activates NG neurons and elucidated underlying mechanisms. Insulin interacted with isolated NG neurons of mouse vagal afferents and induced depolarization and increases in [Ca^2+^]_i_. IR and IRS2 were expressed in NG neurons, and the insulin-induced increases in [Ca^2+^]_i_ were suppressed by blockade of IRS2, PI3K, L-type and N-type Ca^2+^ channels. More than half of the NG neurons that responded to insulin were immunoreactive to CART. Furthermore, some of the neuronal fibers in and around pancreatic islets were immunoreactive to IR. Specific subpopulation of NG neurons that innervate the pancreas, identified by retrograde tracer DiI, responded to insulin with greater incidence than the rest of NG neurons. The insulin concentration in the pancreatic vein was found to be much higher (around 2 log orders), particularly under fed conditions, than that in circulation, and insulin in this high range (>10^−7^ M) recruited a remarkably greater population of NG neurons to [Ca^2+^]_i_ increases. These results suggested that NG neurons serve as a sensor of insulin released in the pancreas. This possibility was also supported by the result that systemic administration of sulfonylurea rapidly induced insulin release and activated insulin signaling, phosphorylation of AKT, in NG neurons. These data collectively demonstrate that insulin directly activates vagal afferent neurons including those containing CART and those innervating the pancreas via IR signaling cascade and depolarization-gated Ca^2+^ influx. Our finding also supports that NG neurons could sense the change of insulin concentration in the pancreas in response to food intake and upon challenge with insulin secretagogues, and subsequently inform the brain of the pancreatic and peripheral insulin changes associated with nutritional states.

Blockade of IRS2 and PI3K in NG neurons decreased the incidence of [Ca^2+^]_i_ responses to insulin to approximately 60∼70% of the control. The results indicate that IRS2 and PI3K are mandatory required for insulin to increase [Ca^2+^]_i_ in some of NG neurons. Furthermore, in the rest of NG neurons in which [Ca^2+^]_i_ increases remained under blockade of IRS2 and PI3K, the amplitudes of [Ca^2+^]_i_ increases were markedly suppressed to 30∼45% levels. These data demonstrate that the insulin signal cascade of IR-IRS2-PI3K plays an essential role in the responses to insulin in NG neurons, though other pathways may also be partially involved in some of insulin-responsive NG neurons.

In NG neurons, the resting membrane potential of around -50 mV was depolarized to around -30 mV by insulin. The insulin-induced increases in [Ca^2+^]_i_ were largely and partially inhibited by blockers of N- and L-type Ca^2+^ channels, respectively. These results indicate that insulin depolarizes NG neurons and consequently activates voltage-gated Ca^2+^ channels, predominantly N-type and additionally L-type channels. A key role of N-type Ca^2+^ channels was previously shown for the nesfatin-1-induced [Ca^2+^]_i_ increases in NG neurons [14].

It is thought that the terminals of vagal afferents innervate peripheral organs and sense the local information in and around the organs [19]. For instance, it has been reported that CCK at high concentrations, which correspond to the estimated levels of CCK in the duodenum and jejunum where this hormone is released, activates NG neurons innervating the upper gastrointestinal tract in a paracrine manner [13]. Vagal afferent neurons reportedly also innervate the pancreas [21], [22]. In the present study, we showed that some of neuronal fibers innervating the pancreatic islets expressed IRs. Moreover, we have established the method to selectively analyze the NG neurons innervating the pancreas, by specifically labeling them with the retrograde fluorescence tracer DiI injected into the pancreas. We found that the NG neurons innervating the pancreas, identified by DiI fluorescence, responded to 10^−7^ M insulin with [Ca^2+^]_i_ increase at a three-fold higher incidence (20%) than unlabeled neurons. This result suggests that the NG neurons innervating the pancreas are highly equipped with insulin-responsive machinery, which might serve to quickly and efficiently sense insulin at the place where it is released. This paracrine mode of action often requires higher concentrations of hormones than the endocrine action. It has been reported that insulin released from pancreatic β-cells acts on other cells in the pancreas in a paracrine manner [23], [24], and that the insulin concentration around islet β-cells during stimulation with high glucose is estimated to be 100∼200 nM [25], a level at least 1,000-fold greater than systemic concentration of insulin [26]. In the present study, insulin concentration in the pancreatic vein under *ad lib* fed condition was over 10^−8^ M in rats, and the insulin concentration in/around islets is speculated to be even higher. It was reported that the estimated transient peak level of insulin around islets after meal intake is 5∼6 times higher than the insulin level in *ad lib* fed state [27]. We found that the elevation of insulin concentration from 10^−10^∼10^−8^ M to 10^−7^ M recruited a remarkably greater population of NG neurons (approximately 12% with 10^−7^ M vs. 3% with 10^−10^∼10^−8^ M insulin, Fig. 1D). Therefore, it is suggested that the responses of NG neurons to 10^−7^∼10^−6^ M insulin might reflect the *in vivo* condition that insulin released in the pancreas at high concentrations after meal intake activates NG neurons that innervate pancreas in a paracrine manner. On the other hand, the responses to 10^−10^∼10^−9^ M insulin might reflect the *in vivo* condition in which NG neurons respond to circulating insulin. The mechanism underlying the concentration-dependent two-step activation of NG neurons by insulin remains to be elucidated. However, since we confirmed the mRNA expression of IGF-1 receptors in NGs (data not shown), the interaction of insulin with insulin receptors and IGF-1 receptors could be involved in the two-step activation.

In this study we found that insulin action on NG neuron was markedly impaired in IRS2-KO mice. Hence, this animal could provide a good model to assess the physiological role of NG neuron-mediated insulin sensing and information transmission to the brain. In this study we found that insulin action on the first order hypothalamic neurons was intact, while that on NG neuron was markedly impaired in IRS2-KO mice. The results suggest that hyperphagia and obesity, disorders of the primarily central origin, observed in IRS2-KO mice [28] and neuron-specific IRS2-KO mice [29], could be, at least partly, due to impaired ability of NG neuron to sense insulin and thereby convey its information to the brain in IRS2-KO mice. It was previously reported that knockout of IRS2, but not other subclasses of IRS, exhibits hyperphagic and obese phenotypes, indicating an important role of IRS2 in regulation of feeding and body weight [30]. Therefore, the IRS2-dependent insulin reception by NG neuron, revealed in the present study, could be implicated in the regulation feeding and body weight (Fig. 6D).

The NG neurons sense and convert peripheral chemical signals to neuronal impulses which are conducted to the nucleus tractus solitarius (NTS) of the brainstem. For instance, the activation of vagal afferents by peripheral CCK leads to c-fos expression in the NTS neurons [31], [32]. It is therefore suggested the activation of NG neurons by insulin could be followed by neurotransmission in the NTS. In line with this, Yan A. et al. reported that peripheral injection of insulin in euglycemic conditions induces c-fos expression in the NTS [33]. The NTS neurons project to various brain regions including the hypothalamic arcuate nucleus and paraventricular nucleus, the pivotal feeding centers regulating feeding, metabolism and autonomic nerves [34]. Furthermore, in our study, the NG neurons that responded to insulin always responded to capsaicin. It was reported that the capsaicin-sensitive NG neurons mediate the effects of GI hormones including CCK, glucagon-like peptide-1, peptide YY, leptin, and ghrelin on feeding, gastric motility and insulin release [35]–[43]. Moreover, we found that more than 50% of insulin-responsive NG neurons were immunoreactive to CART. It has been reported that anorexigenic CCK1 receptor is expressed in CART-immunoreactive NG neurons [20], and that expression and secretion of CART is stimulated by CCK-8 in NG neurons, suggesting an anorexigenic neurotransmitter role of CART in NG neurons [44], [45]. Taken together, the insulin activation of capsaicin-sensitive NG neurons containing CART could be implicated in the regulation of feeding and metabolism. Further studies are definitely required to determine whether the insulin action on vagal afferent is relayed to stimulation of the NTS and hypothalamic neurons and to regulation of the brain and systemic functions, including feeding and metabolism.
